# Annotations

**Published:** 1898-11-12

**Authors:** 


### ANNOTATIONS.

### The Plague Commission.

The Government has done well in appointing a strong Commission to proceed to India to report upon the plague. The scope of the Commissioners' inquiries will include (1) the origin of the different outbreaks of plague; (2) the manner in which the disease is communicated; (3) the effects of certain prophylactic and curative serums that have been tried or recommended for the disease. It is difficult to exaggerate the importance of the work set before the Commission, especially in regard to the last subject in the reference, for it goes out at a moment when the efforts to control plague by isolation and segregation, although tried on the most lavish scale, and enforced with a display of military power unprecedented in countries under English rule, have ended in complete failure, and when consequently a strong tendency is manifesting itself to a revulsion to inoculation. It is impossible, then, not to see that we stand, as the saying is, at the parting of the ways, and that the conclusions of this Commission are likely to have much influence in setting the fashion of the immediate future in regard to the management of other infectious diseases besides plague. With the exception of the control of small-pox by vaccination, the official view of English sanitarians has long tended in the direction of lessening the virulence, not of one disease, but of all diseases, by improving the conditions of life, and of treating "outbreaks" by the isolation of those who are actually affected. Under the influence of this tradition England has done well, and any proposal to protect individuals by inoculation instead of protecting communities by sanitation should be looked at with great caution. An article last week in the *Times* and an Editorial in this month's *Public Health* show clearly enough how strong is the present drift towards inoculation, and hence how important is the work, we may almost say the decision, delegated to this Commission. For this reason we should have been glad to have seen upon it some level-headed representative of the accumulated scientific experience of the Local Government Board; for, although we have every confidence in the scientific members of the Commission, we cannot but remember how largely "bacteriology" and "immunity" figure in the work for which they are best known.

### Long Heads and Thick Heads.

In the last number of *Science Progress*, which we regret to find is to be really the last number that will be published, there is an interesting article by Beddoe on "Selection in Man," which throws a somewhat curious light upon the influence which is exercised on the race by emigration and by the intermarriage which goes on between communities. Roughly, the inhabitants of Europe may be divided into long heads and broad heads—thick heads, if one likes so to call them. This division is one which anyone can verify, it is a mere matter of measurement, and of the relation between head-length and head-breadth. The interest of the fact, however, lies in this, that these two types of head are associated with two distinct types of man, distinct in their intellects, their tendencies, and their ambitions. The long heads are the salt of the earth; the stirring, active, ambitious, independent, courageous, locomotive element of mankind; and "it appears to be this character, which is connoted by that projection of the occiput, which is more common to the inhabitants of these islands than in those of almost any other country." The broad head, on the other hand, "is frugal, laborious, or at least economical, remarkably prudent, and though not cowardly, not warlike. His intelligence is usually mediocre, and he works out patiently his limited ideals." The adventurers, those who are not content to pass their lives as they were born, who do not seek their wives in their own village, and finally who emigrate and crowd into the towns, are the long heads. Thus it is not the whole of the country population which gravitates towards the towns. It is on the long heads that the attractions of city life chiefly tell, so that an over large proportion of the broad heads is always left to stagnate in the country. Nevertheless, although most of the great things in the world have been done by the long-head, both he and his progeny is very apt to perish. "How few descendants can be found of great soldiers, travellers, discoverers, inventors, poets. The higher and more enlightened classes in communities, the producers, and assimilators of new ideas, have repeatedly in the course of history been swept away or decimated, while the proletariat survive."

### A Very Old Recipe.

The *Westminster Review* has lately published an article by Lady Florence Dixie on what she calls a "New Gospel of Health." She seems to have been told by some American physician that all disease is the result of eating in excess of the supply of gastric juice, and on that she founds the doctrine that we can keep well by eating only when we are hungry, and by then only eating sufficient to satisfy that hunger. And if all disease were located in the stomach, and if people could live on without food until their disease had done with them and left them free to enjoy that hunger which can be cultivated "by the virtue of abstemiousness," then there would be a certain amount of truth in this "gospel." But it would not be "new." The preaching of abstemiousness is the daily and thankless task of every doctor in the land; and "to live on sixpence a day, and earn it,' is an old prescription that descends to us from one of the masters of medicine. The one end of the prescription, however, is as true as the other, and for the cases in which it is beneficial the earning the sixpence is almost as important as the living on it. Such generalising from individual experience as leads to this sort of article is the common fault which overhangs all amateur doctoring. When a lady chooses to proclaim a treatment which *will never fail*, all sensible people know what to think of it; and our only reason for alluding to the subject is to warn people not to play tricks with the stomach even on the pretence of giving it rest.

